# Treatment by Light and X-Rays

**Published:** 1901-10-05

**Authors:** 


### TREATMENT BY LIGHT AND X-RAYS.

The experience which has now been gained in the use of light and X-rays in the treatment of lupus and some other diseases enables one to make some sort of estimate as to the cases in which these agents are likely to do good. During the meeting of the British Medical Association many observers interchanged opinions on the subject.

Up to July of this year Mr. Malcolm Morris had treated 60 cases by light. In 36 of these the disease was lupus vulgaris, and of these eight might be claimed as instances of cure; in three the treatment was abandoned on account of failure of health, while in six the result was unsuccessful or unsatisfactory. The remainder were still under treatment. The cases of rodent ulcer numbered 14, and of these seven were cured; in two it was impossible to go on with the treatment because of the unsatisfactory state of the patient's health, while two were still under treatment. Dr. Sequeira, speaking from the experience gained at the London Hospital, where nearly 200 patients had been under treatment, said he had observed improvement in every case of lupus vulgaris. Cases of every degree of severity had been under treatment. The number of applications which might be necessary varied greatly, for, while in two cases, where the disease was quite recent, two applications had resulted in the absolute disappearance of the lupus, in other and more extensive cases 200 and even 300 sittings had been given and the patients were still under treatment. Mr. Morris had pointed out the same thing, the number of sittings in his successful cases having varied from eight to more than 370. In regard to the choice of cases favourable for the light treatment, Mr. Morris said that a thin skin was favourable, a coarse skin being less easily penetrated by the light. The amount of pigment naturally present in the skin must also be taken into account. Brunettes were not such good subjects as blondes, and the darker they were the less amenable were they to the light treatment. Again, when the disease extended deeply and there was much thickening and infiltration of the skin, it was difficult for the light to penetrate, and in the elephantiasis-like forms of lupus sometimes seen on the ears, cheeks, and elsewhere the reactions might be so severe that they could not be borne by the patients. A large area of disease was a most serious difficulty, as only a small portion could be treated at a time, and it was difficult for the treatment to keep pace with the progress of the disease. Cases affecting mucous membrane were unfavourable for the light treatment alone, but in these cases the X-rays were of great service. Other things being equal, cases which have not been much treated would seem to be more favourable than those which have already been dealt with by cauterisations and scrapings, the effect of such treatment being in some cases not only to render the skin almost impenetrable to light, but also to make it difficult to apply the pressure-glass.

